# American Veterinary College

**Published:** 1893-12

**Authors:** 


AMERICAN VETERINARY COLLEGE.

*March 24th.—Degree D. V. S. (Doctor of Veterinary Surgery.)*

| | |
|---|---|
| William Alexander Attfield - - | Brooklyn, N. Y. |
| Leo Edward Buckley - - | New York, N. Y. |
| Charles Reuben Biederman - - | Brooklyn, N. Y. |
| Harry H. Bear - - - | Mount Joy, Pa. |
| Alexander Joseph Burkholder - - | Staunton, Va. |
| Grantley Willoughby Bickell - | Haverhill, Mass. |
| Charles Edward Clayton - - | Waltham, Mass. |
| Charles Henry Doepel - - | Mamaroneck, N. Y. |
| Frank P. Dorian - - - | Yonkers, N. Y. |
| William Freeman Davis - - | Washington, C. H., O. |
| John Archie Eadie - - - | New Brighton, N. Y. |
| William C. Ferguson - - | New York, N. Y. |
| Charles Edgar Garman - - | Nora Springs, Iowa |
| Samuel Glasson, Jr. - - | New York, N. Y. |
| John Henry Gardner, Jr. - - | Norwich, Conn. |
| Louis Henry Hempelman - | St. Louis, Mo. |
| Gottfried Leonhard Hagenburger | Hettenheim, Germany |
| Harry Newel Hall - - | New Haven, Conn. |
| Mark Edward Johnson - - | Red Oak, Iowa |
| Leander Young Ketcham, Ph.G., M.D. | Woodbury, Conn. |
| Samuel Erdman Lloyd - - | Govanstown, Md. |
| Edward Bedell Metcalf - - | Albany, N. Y. |
| Albert Francis Mount - - | Jersey City, N. J. |
| Charles Henry Martin - - | Dobbs Ferry, N. Y. |
| John J. Marshall - - - | New York, N. Y. |
| Henry Joseph McClellan - - | Bryn Mawr, Pa. |
| William Townsend McCoun - - | Oyster Bay, N. Y. |
| John Peter Nestler - - | Jersey City, N. J. |
| Charles Allen Parkerson, Jr. - - | New York, N. Y. |
| John Vernon Prather - - | Troy Centre, Pa. |
| James A. Peed - - | New Castle, Ind. |
| William Avin Porter - - | Dunksburg, Mo. |
| Rudolph Bertram Plageman - | Brooklyn, N. Y. |
| Joseph Bennett Quin - - | Cincinnati, Ohio |
| Charles Albert Raque - - | West Nyack, N. Y. |
| Napoleon Bonaparte Rhodes - - | Brookville, Fla. |
| Warren Lawrence Rhoades - - | West-town, Pa. |
| John Edward Rowe, Jr. - - | Newark, N. J. |
| Raymond Brooks Smith - - | Montclair, N. J. |
| Harry Frank Steele - - | Titusville, Pa. |

| | | |
|---|---|---|
| Isam Enoch Scripture | - - | Frankfort, Ind. |
| Harry Edward Styer | - - | Medina, Ohio |
| Charles Schroder | - - - | Brooklyn, N. Y. |
| William Prout Straughan | - - | Jewett, N. Y. |
| Shirley Bruce Staples | - - | Alexandria, La. |
| Robert Smith Todd | - - | Waterbury, Conn. |
| Howard Stanton Usher | - - - | Hollis. Me. |
| Ernest Lewis Volgenau | - - | New York, N. Y. |
| Charles Lucian Van Schaick | - - | Brooklyn, N. Y. |
| Samuel Adam Wright | - - | Long Island, N. Y. |
| Richard Mitton Weightman | - - | Utica, N. Y. |
| Frank Potts Williamson | - - | Raleigh, N. C. |

